# Co‐Design of a Registry‐Based Tailored Follow‐up Service Intervention for People Living With Stroke: A Multiple Method Consensus Approach

**DOI:** 10.1111/hex.70474

**Published:** 2025-10-29

**Authors:** Tara Purvis, Andrew G. Ross, Jannette M. Blennerhassett, Karen M. Barclay, Tanya Frost, Dana Wong, Susan Hillier, Kathleen L. Bagot, Joosup Kim, Jennifer Cranefield, Katherine Jaques, Mark R. Nelson, Grant Russell, Colin Scott, Melita Stirling, Monique F. Kilkenny, Natasha A. Lannin, Timothy J. Kleinig, Rohan S. Grimley, Julie L. Morrison, Sandy Middleton, Vincent Thijs, Adele K. Gibbs, Dominique A. Cadilhac, the A‐LISTS investigator group

**Affiliations:** ^1^ Stroke and Ageing Research, Department of Medicine, School of Clinical Sciences at Monash Health Monash University Clayton Victoria Australia; ^2^ Stroke and Critical Care Research theme Florey Institute of Neuroscience and Mental Health, University of Melbourne Heidelberg Victoria Australia; ^3^ Institute for Health & Sport Victoria University Melbourne Victoria Australia; ^4^ Physiotherapy Department and Health Independence Program Austin Health Heidelberg Victoria Australia; ^5^ Care Economy Research Institute La Trobe University Albury‐Wodonga Victoria Australia; ^6^ Department of Neuroscience Eastern Health Melbourne Victoria Australia; ^7^ School of Psychology and Public Health La Trobe University Bundoora Victoria Australia; ^8^ IIMPACT, Allied Health and Human Performance University of South Australia Adelaide South Australia Australia; ^9^ Department of Neurology Royal Adelaide Hospital Adelaide South Australia Australia; ^10^ Wesley Mission Queensland Brisbane Queensland Australia; ^11^ Clinical Excellence Queensland Queensland Health Brisbane Australia; ^12^ Menzies Institute for Medical Research University of Tasmania Hobart Tasmania Australia; ^13^ Department of General Practice, School of Public Health and Preventive Medicine, Faculty of Medicine, Nursing and Health Sciences Monash University Melbourne Victoria Australia; ^14^ Stroke Association of Victoria Melbourne Victoria Australia; ^15^ Enhancing Stroke Care Collaborative Safer Care Victoria Melbourne Victoria Australia; ^16^ Stroke Foundation Melbourne Victoria Australia; ^17^ Department of Neuroscience, School of Translational Medicine Monash University Melbourne Victoria Australia; ^18^ School of Medicine and Dentistry Griffith University Birtinya Queensland Australia; ^19^ Sunshine Coast University Hospital Birtinya Queensland Australia; ^20^ Nursing Research Institute, St Vincent's Health Network Sydney and School of Nursing, Midwifery and Paramedicine Australian Catholic University Sydney New South Wales Australia; ^21^ Department of Neurology Austin Health Heidelberg Victoria Australia; ^22^ Melbourne Medical School University of Melbourne Melbourne Victoria Australia

**Keywords:** clinical registry, co‐design, consensus, Delphi survey, stroke

## Abstract

**Background:**

Often people experience ongoing health challenges after stroke. The Australian Stroke Clinical Registry collects patient‐reported outcomes after stroke. Many patients report challenges that are potentially addressable through additional support.

**Aims:**

To co‐design a registry‐based, hospital‐initiated, follow‐up service for people who report major health‐related challenges between 90 and 180 days after their stroke.

**Methods:**

Iterative, consensus‐based methods were used to co‐design a follow‐up service intervention including eligibility criteria, clinical protocol (consultation/communication forms and pathways) and implementation requirements (e.g., training manual) (May 2022–March 2023). Stakeholders, including Australian‐based clinicians providing stroke care, researchers and people with lived experience of stroke, were involved in each stage. Data collection: Stage 1 (development), (i) scoping survey; (ii) two consensus meetings; (iii) interviews with key informants (*n* = 3); (iv) online modified Delphi survey; Stage 2 (testing and finalisation), (v) piloting of the follow‐up service intervention at one hospital, with service coordinator/study team interview and participant satisfaction surveys and; (vi) final review (modified Delphi survey). Consensus was defined in the modified Delphi surveys as ≥ 80% ‘agreement’ or verbal consensus via open voting during meetings. Additional recommendations from each step were iteratively incorporated to refine the intervention.

**Results:**

Scoping survey results (*n* = 41/108 respondents, 38% response rate) highlighted the need for broad inclusion criteria and the involvement of carers/support person and general practitioners. During the consensus meetings (16/18, 89% stakeholders attended at least one), verbal consensus was achieved for the eligibility criteria, and additional recommendations were made for the referral report and components within the clinical protocol and training manual. After the final Stage 1 modified Delphi survey (*n* = 10, two cohorts), 70%–100% consensus was achieved for the referral report, clinical protocol components and training manual, which were then piloted with six eligible participants. Feedback from the pilot testing (*n* = 3 coordinator/staff interviews; *n* = 5 satisfaction surveys) led to further clinical protocol modifications. Agreement was reached for all additional recommendations during the final modified Delphi survey round (16/29 respondents, 55%).

**Conclusion:**

We describe an iterative, consensus‐based co‐design process which resulted in a novel, registry‐based follow‐up service intervention for people living with stroke reporting major health challenges. A feasibility randomised controlled trial is the next stage.

**Patient or Public Contribution:**

People with lived experience of stroke, including their family/caregivers, actively participated throughout the co‐design process to develop and test the follow‐up service intervention. There was lived experience representation with scoping survey responses, as well as within the working group and independent review group who were involved with the consensus meetings and modified Delphi process. Survey feedback from people with stroke who piloted the developed service intervention was also integral to informing the final service intervention.

## 1 Introduction

People living with stroke often face significant challenges transitioning beyond the acute hospital and early rehabilitation phases of recovery. Systematic review evidence from 19 eligible studies suggests that after stroke, 74% of people have a least one unmet need, most commonly related to fatigue, cognitive, emotional and secondary prevention needs [1]. Evidence from the Australian Stroke Clinical Registry (AuSCR) has repeatedly indicated that approximately one in two people with stroke report impacts to various domains of their quality of life between 90 and 180 days after admission [2, 3]. Challenges navigating ongoing care services and not knowing where to find necessary information can compound these difficulties for people living with stroke [4]. Additional barriers to addressing these challenges are exacerbated by current outpatient services for stroke often not being targeted to the people who report the most need [5], a lack of timely and coordinated approaches by health service, and challenges identifying those with the most needs. Therefore, effective solutions to identify, refer and support people living with stroke in the community with health challenges or activity limitations are required.

The transition from hospital services to primary care represents a vulnerable time for people with stroke and their family/carers, when dealing with stroke‐related problems [6]. Integrating care through a coordinated and collaborative approach between hospital and primary care settings is essential for enhancing patient outcomes, improving care efficiencies and reducing costs [7, 8]. In Australia, the median length of stay after acute stroke is only 5 days, with approximately one in four stroke survivors discharged to inpatient rehabilitation. Of the half who return directly home after acute care, around 20% have no support in place [9]. Follow‐up review after stroke is recommended with both a general practitioner (GP) and a specialist outpatient clinic [10]. However, increasing demand has made timely GP access difficult [11] and only 30%–50% of patients are seen in neurology clinics within 90 days post‐stroke [5]. The Stroke Foundation also offers additional support through StrokeLine (telephone advice—StrokeLine | Stroke Foundation—Australia) and EnableMe (online recovery tools and peer support—Home | enableme—stroke recovery and support), although awareness and integration with existing care pathways remain limited.

Authors of a recent prospective observational study evaluated a neurological nurse‐led model of post‐discharge care that produced cost savings, improved functional status and improved health related quality of life [8]. Similar benefits were observed in a structured post‐stroke care coordination programme delivered through both telephone and face‐to‐face contact, with a reported four‐fold increase in outpatient service use and a 58% reduction in hospital readmissions and associated costs in the first year compared to the control group [12]. While these studies demonstrate that improved collaboration between hospital and primary care settings yields significant benefits for those living with the effects of stroke and other chronic conditions, additional difficulties exist in identifying unmet health needs that could be addressed with such services.

Patient‐reported outcome measures (PROMs) capture a person's perception of their own health to inform clinicians about aspects of the person's well‐being that are important. Although PROMs data are rarely collected by hospitals or primary care services, the AuSCR collects this information from all eligible participants between 90 and 180 days post‐admission [2]. These data can potentially support the identification of ongoing challenges or unmet health needs of people with stroke in the community and provide evidence for designing solutions in a fragmented health system that can be difficult to navigate.

Using co‐design alongside consensus methods offers a powerful approach to developing relevant interventions or services [13]. The combination of active involvement of expert stakeholders in the design process with structured decision‐making to iteratively refine a service or intervention provides both inclusivity and agreement. This process ensures that the final solution is more likely to be both practical and widely supported [14].

The aim of this study was to co‐design a registry‐based, hospital‐initiated follow‐up service intervention for people who report health‐related challenges or difficulties affecting physical, mental or social well‐being, between 90 and 180 days after their stroke.

## 2 Methods

We adopted a staged co‐design approach [15] informed by participatory action research methodology [16] and iterative consensus methods to develop the registry‐based, hospital follow‐up service intervention. We involved different stakeholders including clinicians, patient or policy advocates, researchers, and people with stroke or caregivers as lived experience representatives. All lived experience representatives received vouchers covering sitting fees of $50/hour for their time in contributing to the study.

The study was designed to integrate both quantitative and qualitative consensus methods [17] when obtaining stakeholder feedback to inform the development and refinement of the follow‐up service intervention (Stage 1). The final prototype was subsequently pilot tested at one hospital (Stage 2). Feedback from the pilot stage was then incorporated to finalise the intervention for testing in a future feasibility randomised controlled trial. Clear roles and cooperation between the registry and hospital were fundamental to the model of care being proposed.

**Figure 1:**
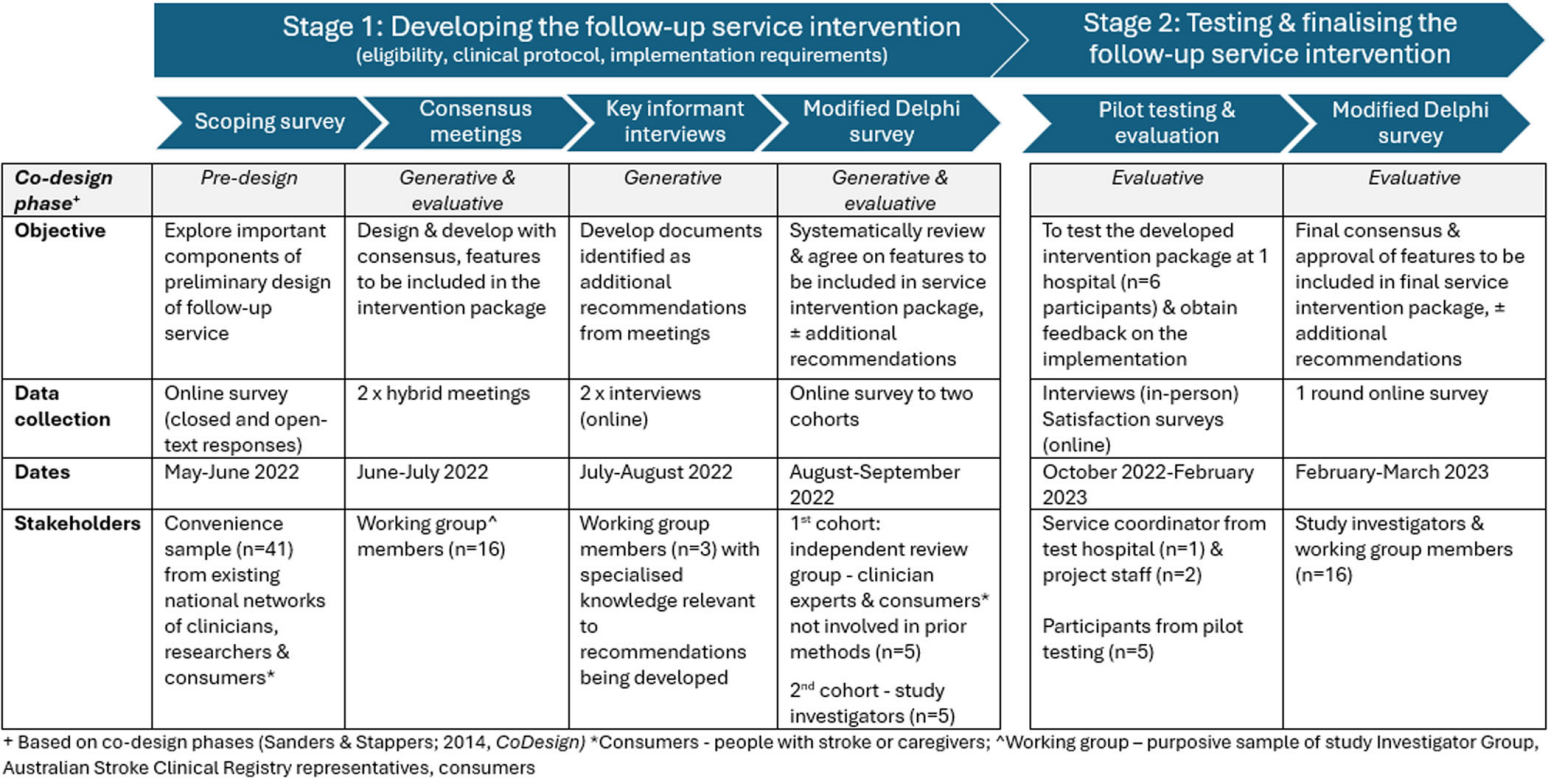
Overview of the co‐design and consensus methods.

Specifically, the consensus‐based approaches included data collected from (i) a scoping survey, (ii) stakeholder consensus meetings including open voting [17], (iii) interviews with key informants, and (iv) online modified Delphi survey including two separate cohorts. Following the pilot testing of the developed intervention at a single hospital, (v) test participants completed an online survey and a reflection interview was held with project staff and the service coordinator and investigators (D.A.C. and A.R.) to consider further refinements and improvements (Figure 1). Further details are provided in the description of the following Stages 1 and 2.

The ACCORD (ACcurate COnsensus Reporting Document) guideline was used for reporting the methods and results of the consensus aspects [17] with reference to the Consolidated Criteria for Reporting Qualitative research (COREQ) for qualitative components [18]. A protocol for the co‐design process was not prospectively registered. However, ethics approval was obtained before data collection (HREC/86507/Austin‐2022).

The study investigator group, which included national leaders with experience in undertaking successful multisite, interdisciplinary complex healthcare projects, had conceptualised a preliminary design for the follow‐up service intervention as part of securing grant funding. This conceptual design encompassed three main components including: (i) identifying eligible participants from the AuSCR [19] and communicating with hospital staff; (ii) the clinical protocol to be delivered by the hospital‐based service coordinator; and (iii) service implementation requirements (Table 1).

**Table 1:** Preliminary design of the follow‐up service intervention.

Component	Description	Features of service for co‐design and consensus
Identify eligible participants and communicate with hospital staff	AuSCR registrants whose responses to 90–180 day outcome survey met the agreed eligibility criteria to be identified AuSCR Data Manager to notify hospital staff of eligible participants using a secure method	Eligibility criteria Method of notification and to whom (at hospital) Referral: report type and what information should be included in the referral correspondence
Clinical protocol for the follow‐up service to be delivered by the hospital‐based service coordinator	Guidance document for delivering the hospital‐initiated follow‐up service intervention (that can be tailored based on local resources and requirements)	Initial consultation form (template for initial conversation) to be completed by the service coordinator Specific consultation pathways (outlines potential service steps and activities—three options identified initially)
		– No further action required after initial consultation – Further action by service coordinator – Uncontactable/refusal to engage
		Referral/communication forms (for participants and general practitioners)
Implementation requirements	Service coordinator identification Training tools/manual to support standardised delivery Hospital specific processes/policies for consideration	Identification of important characteristics for the hospital‐based service coordinator delivering the intervention Training procedure manual for service coordinator considering needs and requirements

*Note:* Modifications to the intervention as a result of the co‐design process are depicted in Tables 3, 4 and 5. Service coordinator—hospital clinician delivering the follow‐up service.

Abbreviation: AuSCR, Australian Stroke Clinical Registry.

### 2.1 Stage 1: Developing the Follow‐up Service Intervention

#### 2.1.1 Scoping Survey

An initial online scoping survey was undertaken to explore stakeholder perspectives about important features to be included as part of the proposed follow‐up service and to obtain feedback on the preliminary design components of the intervention. The survey was developed by the project team incorporating stroke care expertise and relevant literature considerations. Initial pilot testing was undertaken with four people representing the target population (lived experience representatives, clinicians and researchers), with subsequent refinement based on their feedback before distribution. The final survey included multiple choice and open‐text questions about the content and logistics related to the proposed components and features in the intervention such as: (i) the criteria to identify people living with stroke who may benefit from a follow‐up service; (ii) how the AuSCR could notify hospital service coordinators about eligible registrants; (iii) steps to be undertaken by the service coordinator in delivering the service (e.g., clinical protocol) and (iv) other requirements for implementation of the service, for example, training materials or background required for a service coordinator, and local hospital processes/policies for consideration (Table 1) (Supplemental Materials). Open‐text questions were included to capture perceptions on additional factors to consider in a follow‐up service for people post stroke. Preliminary clinical protocol details and proposed associated documentation were embedded in the online survey allowing respondents to access these illustrative examples as they progressed through the questions.

A convenience sample was drawn from established national networks of the study investigators within Australia. Members of the investigator team contacted potential participants via email and/or phone and invited them to complete the scoping survey. This sample included clinicians involved in stroke care from general practice and community settings, as well as representatives from hospitals contributing to the AuSCR (clinicians and managers), patient advocate organisations (e.g., Stroke Foundation and Stroke Association of Victoria) and lived experience representatives.

The self‐reported survey was delivered in an online electronic format (created using Qualtrics software © 2023 Qualtrics, Provo, Utah, the United States). Participants were sent a personalised email with a general link to the survey to ensure anonymity was maintained. No identifying information was collected unless respondents wished to receive a summary of results. In such cases, this information was stored separately to survey responses, with de‐identified data provided for analysis. At least one reminder email to complete the survey was provided. The survey was open for 3 weeks (May to June 2022). Completion of the survey was considered implied consent.

We used descriptive analysis to summarise the closed questions within the scoping survey. Open‐text responses for each component were subjected to directed qualitative content analysis [20]. Two researchers (A.R. and K.M.B.) independently coded responses, using a deductive approach based on the main components of the preliminary service intervention. They systematically identified categories that reflected similarities and/or differences in respondents' viewpoints in relation to these components. Discrepancies were resolved through discussion to reach consensus. This approach enabled structured interpretation of participant responses concerning the main components, while also allowing for the identification and inclusion of additional insights where applicable. The findings from the scoping survey guided discussions in the consensus meetings around specific features to include in the follow‐up service intervention.

#### 2.1.2 Consensus Meetings

We held two consensus meetings with a working group 3–5 weeks after the scoping survey closed, to iteratively discuss, adapt and refine the features of the preliminary service intervention components (i.e., eligibility, clinical protocol and implementation requirements). Up to three meetings were originally planned to ensure that all stakeholders could actively contribute and that consensus would be reached on the relevant components of the service intervention. The working group (*n* = 18) included a range of interdisciplinary participants from around Australia, identified through purposive sampling of the study investigator group. Of 23 invited, 18 agreed to participate. This included clinicians and managers from the AuSCR hospital network and wider stakeholders including lived experience representatives and patient advocate organisations (e.g., Stroke Foundation). All working group members had been invited to complete the scoping survey.

Meetings were conducted in a hybrid forum, permitting both in‐person and online attendance. Participation rate of at least 75% of working group members at each meeting was encouraged to ensure representative responses were captured. The consensus meetings were facilitated by D.A.C. (female, PhD, experienced health services researcher) and lasted for 2 h.

Before the first meeting (June 2022), a summary report from the scoping survey was circulated via email. The first meeting focused on introducing the proposed follow‐up service design with summarised results from the scoping survey presented. These results included preferences as to whom at the hospital should receive notification of eligible participants and how and suggested considerations to the service components from open‐text responses. Facilitated discussion during the first meeting focused primarily on determining eligibility criteria (e.g., defining unmet needs) and potential consultation pathways that may occur within the follow‐up service. During the second meeting (July 2022), the group discussed and explored the initial consultation form, key contact form (for communication with participants) and training considerations for the service coordinator.

We measured consensus during these meetings by capturing the collective views of attending stakeholders. This process was completed through structured discussions, followed by summarisation by the facilitator, with stakeholder open voting (mixture of verbal approval and show of hands) and/or confirmation, such as asking, *‘Does anyone have any objections or additional thoughts?*’ Each meeting was recorded with consent, and additional notes were taken during the meeting by study team members (A.R., K.M.B. and a research assistant). As a summary of discussions was undertaken in real time during the meetings, with stakeholders providing feedback throughout, additional analysis was not required [21]. However, the meeting recordings and additional notes were summarised by one researcher (A.R.) in meeting minutes, which were confirmed by stakeholders. Features where consensus was reached were documented, with these suggestions incorporated into the next iteration of the follow‐up service intervention documents. Areas where consensus was not reached, or proposed recommendations and modifications were raised during the meetings (e.g., features that required additional exploration or development), were outlined for further consideration during subsequent consensus steps. These details were included in the minutes for each meeting which were circulated to working group members, including those who were unable to attend, enabling them to provide comment and input.

#### 2.1.3 Key Informant Interviews

We undertook two key informant interviews with nominated stakeholders from the working group who had specialised knowledge relevant to the additional recommendations made during the consensus meetings (July/August 2022). One individual interview was conducted with a GP to develop the GP‐specific referral letter for the follow‐up service. A group interview was also held with a neuropsychologist and physiotherapist, both experienced in developing educational training, to further discuss specifics related to the training procedure manual. Interviews were 45–60 min, conducted online and facilitated by A.R. (male, MPH, study coordinator). Input from these interviews informed the content to be included in the relevant documents. The subsequent recommendations from these interviews were incorporated into the next iteration of the follow‐up service intervention documents circulated for review in the modified Delphi survey.

#### 2.1.4 Modified Delphi Survey: Consensus

The modified Delphi survey was delivered to an independent review group and the study investigators to gain consensus on the next iteration of the follow‐up service intervention documents (August–September 2022). Anonymity was preserved, with the survey delivered online (Qualtrics) via a general link, and was open for 3 weeks. One reminder was sent before the survey closing. The independent review group (*n* = 6), comprising clinical experts and lived experience representatives not involved in the initial design or earlier consensus methods, was established via invitation, with all invitees accepting. They were purposively sampled from existing investigator networks and invited to participate via email and/or phone. The study investigator group (*n* = 16, five also involved in the working group) were also invited to complete the modified Delphi survey. We aimed for a response rate of 70% in each modified Delphi round [22].

The survey incorporated sections related to the interventions' main features including relevant documents for review and a subsequent question to ascertain if participants agreed with the proposed content (yes/no/unsure) (Supplemental Materials). If participants disagreed, were unsure about, or wanted to provide further contextual details related to the content of the feature, an open‐text question was included for further elaboration on recommended adaptations and other factors to be considered.

Consensus was defined in the modified Delphi surveys as ≥ 80% of respondents being in ‘agreement’ [22] with the proposed features outlined in the follow‐up service intervention documents. ‘Unsure’ responses were considered to be non‐agreement. For features receiving < 80% endorsement, any additional recommendations or modifications from open‐text responses were incorporated before the next consensus step. Therefore, no features were removed, rather the focus was on modifying the intervention content or process until consensus was reached. Features with consensus, along with recommended additions or adaptations, were included in the follow‐up service intervention documents to be pilot tested in Stage 2.

### 2.2 Stage 2: Testing of the Follow‐up Service Intervention and Final Review

#### 2.2.1 Pilot Testing

The follow‐up service intervention documents developed in Stage 1, as well as all the planned outcome assessments (e.g., EuroQoL EQ‐5D‐3L [23], Long‐Term Unmet Needs after Stroke questionnaire [24] and participant diary to record health service utilisation) were pilot tested as a 12‐week programme at a single hospital with six eligible participants (October 2022 to February 2023). Specific time frames, such as the interval between contacts, were based on participant needs and local resources. This test hospital was chosen for pragmatic reasons related to location and as the service coordinator had been involved in the development of the intervention. The hospital was a multi‐campus, tertiary teaching hospital with acute, subacute and community services, and strong clinical and research connections located in a metropolitan area in Victoria, Australia. It provided a variety of primary, secondary and specialist healthcare services, with a specialist stroke unit, and inpatient rehabilitation services, as well as home‐based and community rehabilitation. The test‐site service coordinator was an experienced stroke physiotherapist with expertise across inpatient, outpatient and community stroke services. They received approximately 5 h of in‐person training delivered by A.R. (researcher, study coordinator) which focused on the skills and knowledge required to deliver the follow‐up intervention as outlined in the intervention training manual. Feedback on the pilot testing of the developed follow‐up service intervention documents was obtained through (i) reflection interview with the test‐site service coordinator and two AuSCR staff involved in the identification of eligible participants and (ii) satisfaction surveys from participants. The reflection interview (facilitated by D.A.C. and A.R.) explored the experience of delivering the follow‐up service, using a semi‐structured interview schedule. Questions referenced the Normalisation Process Theory [25] and were tailored to the intervention components, to explore how the developed follow‐up service was embedded and integrated into practice at the test site. The participants who received the follow‐up service intervention in the pilot phase were also asked to complete an online satisfaction survey (closed and open‐text responses) to understand their experiences of the service. Analyses of pilot data focused on identifying challenges and recommended service changes or modifications, with qualitative data (interviews and survey open‐text responses) subjected to qualitative content analysis [20] and closed questions analysed descriptively. Suggested changes and improvements from the pilot testing stage were incorporated into the revised intervention documents which were circulated to the working group as part of the final consensus process.

### 2.3 Modified Delphi Survey: Final Consensus and Approval of Intervention Components

A final online (Qualtrics) modified Delphi survey link was circulated by email to the investigator and working group members for consensus and approval (February/March 2023). The survey included: (i) a summary of each modification to the service intervention documents following pilot testing, in addition to the main features of (ii) the tailored electronic referral report (*communication of eligible participants to the service coordinator*); (iii) the initial consultation form (*template used by service coordinator during initial contact)*; (iv) clinical protocol and consultation pathways (*service steps and activities*); (v) communication forms (e.g., key contact form and GP‐specific letter); and (vi) the training procedure manual. Similar to the prior modified Delphi survey, participants were asked if they agreed with the proposed content/modifications, with open‐text options to add additional recommendations if required. Similar consensus criteria to the initial modified Delphi survey were also applied.

## 3 Results

The findings from each stage are described below.

### 3.1 Stage 1: Developing the Follow‐up Service Intervention Documents

#### 3.1.1 Scoping Survey

In total, 41/108 (38%) responses were received for the scoping survey. Eight responses were from lived experience representatives (11 months to 29 years post stroke), 28 from health service clinicians/managers and 5 from clinician/researchers with stroke experience. Clinicians were from various settings, including five from community or primary care settings, with a range of backgrounds (Table 2, Supplemental Table A).

**Table 2:** Stakeholders involved in the various consensus groups and processes.

Stakeholder group	Scoping survey	Investigator group	Working group^a^	Independent review group
	*n* = 41 *n* (%)^b^	*n* = 12 *n* (%)^b^	*n* = 16 *n* (%)^b^	*n* = 5 *n* (%)^b^
**Lived experience representative**	**8 (20)**	—	**1 (6)**	**2 (40)**
– Person living with stroke	8 (20)	—	—	1 (20)
– Carer	—	—	1 (6)	1 (20)
**Consumer/policy advocacy representative** c	—	**3 (25)**	**4 (25)**	—
**Health service clinician/manager**	**28 (68)**	**2 (17)**	**7 (44)**	**2 (40)**
Doctor	5 (12)	—	1 (6)	—
– General Practitioner	2 (5)	—	1 (6)	—
– Neurologist/General Physician/Geriatrician	3 (7)	—	—	—
Nurse/Coordinator^d^	17 (41)	1 (8)	5 (31)	1 (20)
Allied Health	6 (15)	1 (8)	1 (6)	1 (20)
– Physiotherapist	4 (10)	1 (8)	1 (6)	—
– Speech and Language Therapist	1 (2)	—	—	—
– Occupational Therapist	1 (2)	—	—	1 (20)
**Researcher/Academic**	**3 (7)**	**1 (8)**	—	—
**Academic/Clinician** e	**2 (5)**	**6 (50)**	**4 (25)**	**1 (20)**

*Note:* Bolded rows show broad stakeholder groups; sub‐rows detail specific roles or professional backgrounds within each group.

^a^ five from working group also in the investigator group.

^b^ % may not add to 100 due to rounding.

^c^ For example, representative from the Stroke Foundation.

^d^ Stroke coordinator, Clinical Nurse Consultant, Nurse Navigator, Clinical Nurse or Manager.

^e^ Dual clinical and academic role—neuropsychologist, occupational therapist, physiotherapist or doctor.

The main findings of the scoping survey are summarised in Table 3 along with the resultant topics and content that guided discussion during the consensus meetings. In summary, respondents reported that it was important to capture not just people post‐stroke with physical needs, but those who ‘…*have fallen through the cracks*’ and ‘*may have limited support networks, but are keen to engage with services, they just don't know how*’. It was agreed that the service coordinator needed broad stroke experience, access to a wider multidisciplinary team, good communication skills and knowledge of both the local acute and community services available. However, opinions varied as to whether such a service would fit into the acute hospital setting (which facilitated easy access to medical records/staff, but had high workload demands and resource constraints for providing outpatient services or outreach support for patients discharged more than 6 months prior), or a rehabilitation or community setting.

**Table 3:** Summary of findings from the scoping survey.*

Main component	Summary of findings from scoping survey related to service features	Resultant topics and content to be further discussed in the consensus meetings
Identify eligible participants and communicate with hospital staff	*Eligibility criteria*	Proposed eligibility criteria which includes:
	Consideration should be given to participants with physical and mental health challenges, fatigue and those living alone without social supports—proposed eligibility criteria which considers health‐related quality of life measures cover important areas of need identified by respondents	– extreme problem on any EQ‐5D‐3L domain and/or ≤ 60 on Visual Analogue Scale – age, gender and living situation also need to be considered
	*Referral process*	
	Majority (44%) of respondents felt that a tailored, individualised approach was the most appropriate method to communicate/notify hospital team members of eligible participants, which should be delivered electronically	Specific information to be included in the tailored electronic referral report?
Clinical protocol for the service (e.g., consultation forms, service pathways and referral and communication forms to be completed by the hospital service coordinator)	*Considerations in the initial and ongoing consultations*	Important questions to include in the initial consultation form Decide on proposed clinical protocol process and consultation pathways (e.g., review medical history ± consult medical lead at hospital, contact participant, create plan and determine GP involvement, and set up subsequent consultations as relevant)
	Initial consultation form needs to be comprehensive to uncover ‘*hidden disabilities’—*include questions that ascertain how participants are currently coping at home, what are the unique challenges around their unmet needs, what services are presently being used, or have/haven't worked before, do they feel like they are getting better, staying the same or getting worse? Phone and telehealth options should be offered for consultations within the follow‐up service	
	*Communication with participants*	What information should be communicated to the participant after the consultation—‘key contact form’
	Provision of easy to follow/understandable communication tool or form outlining online information and referral phone numbers would be essential to provide participant	
	*Communication pathways with primary care*	What is the role of GPs in the service, and what is the best method of contact/communication?
	Involvement with the participants' GP in the service process was encouraged, however communication methods and specific GP involvement was unclear	
Implementation requirements (e.g., characteristics of the service coordinator, training requirements and wider hospital considerations)	*Service coordinator characteristics and training requirements*	What is essential to include in the service coordinator training? How much detail to include in training procedure manual?
	The service coordinator at each hospital should be an experienced multidisciplinary team member who has ideally worked across acute and community stroke services (with a knowledge of stroke)	
	*Wider organisational and contextual considerations*	
	Perceived barriers to implementing the follow‐up service include the service cost, funding, time and staff resources Need to take into account wider hospital contextual factors when considering if follow‐up service best place in acute, rehabilitation or community setting	

Abbreviation: GP, general practitioner.

* includes quantitative and qualitative results; EQ‐5D‐3L—European Quality of Life 5 Dimensions 3 Level Version [23] (Health‐related quality of life measure).

#### 3.1.2 Consensus Meetings

Two meetings were undertaken to reach consensus on the service intervention documents, with use of subsequent key informant interviews to further develop suggested specific resources. Overall, 16 of the 18 working group members attended at least 1 consensus meeting; 13 (68%) at the first consensus meeting, and 11 (58%) attended the second (Table 2). Verbal consensus was achieved for the eligibility criteria only. Suggested modifications and recommendations to other service intervention features and documents were generated across both workshops (Table 4). Stakeholders highlighted and discussed the need for:

i. a convenient, secure means to receive the tailored electronic referral report. REDCap (Research Electronic Data Capture) [26] was proposed as a solution, allowing provision of information from the AuSCR team to hospital‐based service coordinators, including notifications of new referrals, as well as enabling data collection by service coordinators (e.g., initial consultation details);
ii. enhanced engagement with the person living with stroke's carer and/or next of kin (‘*stroke survivors don't always recognise they have mental health concerns, that is something picked up by carers or their next of kin*’—carer 03);
iii. recognising differences in health literacy and how questions asked by the service coordinator are phrased (‘*using the term “health” may miss some of the well‐being aspects as some participants may not see fatigue and cognitive functioning as a health issue, more of a lifestyle issues. This might help with identifying the hidden problems that may arise if we stick with medical terminology’*—clinician/researcher 06);
iv. inclusion of a summary of the consultation in the key contact form (which should also include useful clinical service information relevant to the participant);
v. suggestions on the proposed interaction between the service coordinator, the wider interdisciplinary team from the hospital and the participant, and the extent of the involvement of the participant's GP (‘….*integrating any [proposed] model with the GP is [important]… this needs to be co‐designed with GP and practice nurses’*—GP 11), more stakeholder guidance surrounding the creation of the training package for service coordinators and content for the training procedure manual, but ideally it should be a ‘*FAQ/useful information document*’ and be minimalist in design (‘*There will be variation in the capabilities of clinicians [as the service coordinator]. Those with advanced skills will likely use their own mechanisms, yet a more dictated method could be better’*—clinician 07);
vi. including general tips in the training package for searching for local community services.

**Table 4:** Features discussed, consensus achieved and modifications raised during the consensus meetings and modified Delphi survey.

Main component	Feature	Consensus meetings (June–July 2022)		Modified Delphi Survey^a^ (August–September 2022)			
				Consensus^b^			Summary of suggested modifications and recommendations
		Consensus reached?	Summary of suggested modifications and recommendations	Independent group (*n* = 5)	Investigator group (*n* = 5)	Overall	
Eligibility and communication with hospital staff	Eligibility criteria	Yes, Verbal consensus	AuSCR registrants who report an extreme problem in any EQ‐5D domain OR have a HRQoL VAS < 60 will be eligible	—	—	—	—
	Tailored electronic referral report *(communication of eligible participants)*	No	Include details of:	Yes (100%)	Yes (80%)	Yes (90%)	—
			– all hospital admissions – change in status since discharge – communication, fatigue and comorbidities – caregiver contact—important to involve				
			Referral report needs to be provided in secure, convenient manner—REDCap proposed as potential solution for data transfer				
Clinical protocol	Initial consultation form *(template for the service coordinator during the initial phone contact with participants)*	No	REDCap to be used as data collection method Add clearer initial introduction script outlining why they are calling Adjust terminology throughout—avoid ‘unmet needs’ and ‘ongoing health issues’ ‘Areas that should be covered’ instead of list of question to permit tailoring during consultation Included GP detail, if actively involved with GP, and involvement of other specialists Clarify NDIS/My Aged Care^a^ involvement Invite carers and next‐of‐kin to be part of the initial consultation—consent for completion by proxy if participant unable	Yes (100%)	Yes (80%)	Yes (90%)	—
	Consultation Pathways *(service steps and potential service coordinator activities)*	No	Add step to contact GP if participants agree Agreement that the processes for recording the consultation in the hospital medical record will be based in individual requirements	Yes (100%)	Yes (100%)	Yes (100%)	Include recommended time frames for each step within the consultation pathway (e.g., time from referral to initial contact)
	Key contact form *(provide participants with summary of activities and plans from contact)*	No	Include summary of initial consultation in key contact form Provide to participant and carer (if appropriate)	No (60%)	Yes (80%)	No (70%)	Identify best method (email/mail) to send form to participant and carer Include carer contact (if not living with participant) Include section to outline next steps Change name from ‘Summary and contacts’ to ‘Consultation summary and key contacts’
	GP correspondence	No	Develop a GP‐specific correspondence letter in collaboration with GP stakeholder	N/A	N/A	N/A	
Implementation requirements	Service coordinator training procedure manual	No	Consider motivational interviewing as a technique (further discussed in Key Informant Interviews) Include practice calls as part of training Include of crisis procedures/difficult call procedures/cultural competency Include tips for searching community services—impractical to provide inclusive list	Yes (80%)	No (60%)	No (70%)	Include more detail of existing resources available for community supports (e.g., Stroke Foundation resources for health professionals—InformMe, and stroke survivors/carers—EnableMe)

Abbreviations: AuSCR, Australian Stroke Clinical Registry; EQ‐5D, European Quality of Life 5 Dimension; GP, general practitioner; HRQoL VAS, health‐related quality of life Visual Analogue Score; N/A, not assessed in survey; NDIS, National Disability Insurance Scheme; REDCap, Research Electronic Data Capture.

^a^ Australian Government programmes providing funds to support people with permanent and significant disabilities.

^b^ Ascertained from question—do you agree with the specific feature outlined, ‘—’ not asked in modified Delphi survey as consensus reached in meetings.

#### 3.1.3 Key Informant Interviews

A draft of the GP‐specific referral letter was developed following the interview with the GP representative from the working group, with preferred methods of providing the letter discussed. The nature of the study was highlighted, with a summary of the initial consultation, subsequent referrals and contact details for the service coordinator. The letter was focused on improving engagement with primary care and ensuring only relevant information was relayed.

Further discussions with the neuropsychologist and physiotherapist during the second key informant interview helped determine the most effective approach for the service coordinator to engage with participants during consultations. Other recommendations included providing enough information in the training manual to ensure the service coordinator approach was structured while still enabling the consultations to be tailored to the individual.

Inclusions from these interviews were incorporated into the next iteration of the follow‐up service intervention documents circulated within the modified Delphi survey.

#### 3.1.4 Modified Delphi Survey

Ten stakeholders completed the survey (independent review group: *n* = 5 [83%], study investigator group: *n* = 5 [31%]). Overall, consensus was obtained for the content and processes related to the referral report, initial consultation form, and the consultation pathways (90%–100%) (Table 4). We achieved 70% agreement for the key contact form and training manual. Recommendations led to additional changes being incorporated, including a suggested 1‐week time frame from referral to initial consultation, and inclusion of additional existing resources in the service coordinator training manual (Table 4).

### 3.2 Stage 2: Testing of the Service Intervention Package and Final Approval

#### 3.2.1 Pilot Testing

Six eligible people who received acute stroke care at the test site consented to participate in the pilot study (67% male, median age 68 years, median time post stroke 166 days). Of these participants, five completed the satisfaction survey, with 100% agreement that the content of the discussions with the service coordinator were relevant and helpful. Data collected from the participant satisfaction surveys and interviews with the service coordinator and registry staff led to numerous minor editorial changes being made to the follow‐up service intervention documents (Table 5). The main modifications focused on improving the usability/design of the intervention documents. For example, the initial consultation form was modified so the coordinator could make notes directly into the REDCap database rather than having to transpose notes at a later stage, and the GP‐specific letter was simplified. Feedback from both the service coordinator and participants indicated that contacts often involved ‘*advice and education*’ for participants without the need for onward referral. As such, a ‘service coordinator only’ option was added to the consultation pathway in the clinical protocol. Additionally, changes to the participant diary, for participants to record health service utilisation during the 12‐week trial period, were recommended. The changes to the diary were mainly formatting or wording changes to improve the clarity for capturing the details to be included. All recommendations were incorporated into the final version of the service intervention documents that were then circulated to the working and investigator groups for final review and approval.

**Table 5:** Summary of the main modifications to the service intervention documents after the pilot testing.

Main component	Feature	Summary of suggested modifications and recommendations
Eligibility and communication with hospital staff	Tailored electronic referral report	Include email of participant in report
Clinical protocol *(to be completed by the hospital service coordinator)*	Initial consultation form *(template for the service coordinator during the initial phone contact with participants)*	Add process for uncontactable participants Modifications to REDCap database—free‐text notes section added for service coordinators to document what occurred during the initial consultation Ensure outcome of the initial contact linked clearly to the consultation pathway and any additional follow‐up required
	Consultation pathways *(service steps and potential service coordinator activities)*	Two new post consultation pathways created
		– guideline for participant's study withdrawal – ‘advice and education from the service coordinator’ only option (indicating no further involvement required)
	GP correspondence	GP‐specific letter further simplified
Implementation requirements *(e.g., characteristics of the service coordinator, training requirements and wider hospital considerations)*	Service coordinator training package	Intervention documents modified to increase usability for the service coordinator, for example, the training package documents were simplified
	Participant diary	Visual formatting changes with increased detail and clearer instructions for use

*Note:* Final intervention is depicted in Figure 2 (and protocol paper) [27].

#### 3.2.2 Modified Delphi Survey (Review and Approval)

**Figure 2:**
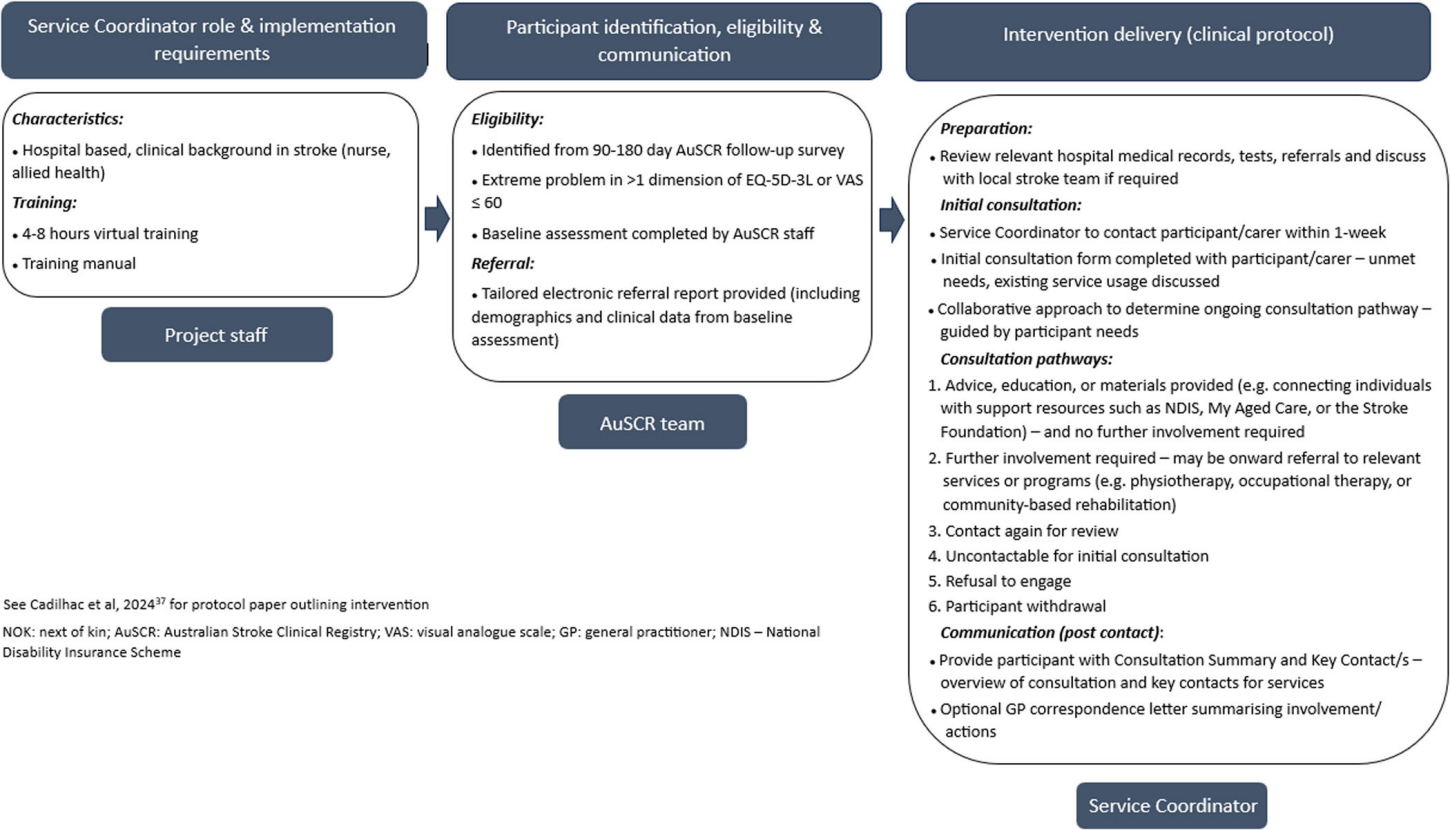
Overview of the final follow‐up service intervention.

In total, 16/28 stakeholders completed the final modified Delphi survey (57% response rate); nine academic/clinicians, two advocacy representatives, one lived experience representative and four health service clinicians. Overall, there was 100% agreement with the modifications made after the pilot testing and with individual features outlined in the final follow‐up service intervention documents and delivery (Figure 2).

## 4 Discussion

We describe our iterative co‐design approach to developing and pilot testing a novel registry‐based, hospital‐initiated follow‐up service intervention targeted to the health needs of people living with stroke. Integral to the process was collaborating with a broad range of stakeholders, including lived experience representatives, researchers and clinicians, and sharing the decision making and responsibility the intervention design [28]. This inclusive approach helped identify specific information related to the eligibility criteria, clinical protocol and implementation requirements which all stakeholders considered to be important for the follow‐up service intervention. The deliberative and consultative engagement of stakeholders [29] throughout the entire co‐design process helped ensure the intervention would be responsive to the needs of the target audience. This comprehensive approach minimised any potential misalignment between different end users and the proposed solution [30] and should increase the likelihood of improving patient outcomes [28].

The use of co‐design methodology for developing stroke interventions is becoming more common. In a 2024 scoping review, Singh et al. identified 89 studies explicitly using co‐design methodology for stroke intervention development. [30]. While prioritisation was acknowledged as an important component of the co‐design process, few authors detailed a structured approach to achieving consensus [30]. This article provides the details of our consensus approach to improve transparency to advance the field. Another advantage was our ability to use the recently published ACCORD reporting guideline [17]. In our study, use of a variety of approaches including a scoping survey, consensus meetings including open voting, interviews with key informants, modified Delphi surveys, and feedback obtained from pilot testing resulted in an intervention design based on successful consensus between lived experience representatives, clinicians and researchers.

Consistent with other consensus‐based studies [31, 32], we found that agreement with specific features increased following each of our consensus steps. Consensus was achieved for all components of the follow‐up service intervention including the eligibility criteria, clinical protocol components and implementation requirements in the final modified Delphi survey. However, an important part of the process was that each step led to inclusion of additional recommendations to ensure the final intervention was fit for purpose. Specifically, the service coordinator and their roles and responsibilities were central to the intervention. This type of specialised role, whereby a skilled professional such as a stroke nurse, physiotherapist or occupational therapist can help coordinate services for a person living with stroke has been proposed as a potential solution to fragmented community care [4, 33, 34]. Linking with the registry allowed our follow‐up service intervention to target people living with stroke more than 6 months after their initial event, a period where ongoing health challenges often affect quality of life. This approach also allows for the identification of individuals who, despite potentially having some initial follow‐up, were experiencing new or ongoing issues at 3–6 months post‐stroke. Given the personalised nature of the interaction between participants and the service coordinator, the intervention may also apply to people at different stages of recovery post‐stroke.

The input received during the co‐design process and feedback from the testing stage aligned with prior research results [4, 35], which indicated that caregivers often feel ill‐equipped and inadequately prepared to care for the person living with stroke at this time period. We therefore modified our follow‐up service intervention to improve engagement with caregivers/next‐of‐kin throughout the process. This included clear requirements in the tailored electronic referral report and initial consultation form which highlighted the need for caregiver engagement.

Stakeholders also reported that communication and interaction between the service coordinator and the participant's GP was necessary, and that developing a structured communication method would be beneficial. This feedback led to having an interview with a GP to develop a GP‐specific correspondence letter as part of the intervention documentation. This outcome illustrates the importance of an action research participatory design [16] for intervention development.

Clinical quality registries have a primary purpose in monitoring the care quality. The AuSCR routinely reports hospital‐level aggregated PROMs collected within 90–180 days, and patient‐level responses are available for hospital staff to view in the registry data platform. However, we are unaware of any hospitals where these data have been actively used to identify and support people with stroke. A major element of this study was the use of AuSCR PROMs data to identify and support people to improve their quality of life post‐stroke once they have returned to the community after being hospitalised for stroke. Using existing outcome data reported from AuSCR, registrants provided a novel means to identify people who may benefit from a follow‐up service without requiring additional data collection. While the participant eligibility identification and referral pathways were satisfactory within the pilot testing, these registry processes will be further assessed as part of the feasibility testing in a multisite randomised controlled trial [27]. The study design also leverages the registry infrastructure reducing resource costs and duplication [36].

Given the depth of experience and expertise drawn on (e.g., through purposive sampling, varied stakeholders and pilot site testing) across multiple complementary methods, we believe we achieved reasonable information power to achieve the aims of this study [37]. However, we acknowledge that involvement from people with lived experience and carers was limited during some phases of the co‐design process, impacted by the Covid‐19 pandemic and study time frames. Nevertheless, all stakeholder groups, including people with lived experience, carers, representatives from advocacy organisations, clinicians and researchers, actively contributed to shaping the programme elements. Notably, no major differences in perspectives emerged between groups, and the collaborative process fostered a shared understanding and consensus across diverse perspectives. Although people with lived experience were remunerated for their time in line with Australian best practice [38], we believe it did not influence their response or engagement with the study. Further engagement with people with lived experience and carers is a key component of the ongoing process evaluation embedded within the feasibility randomised controlled trial. While our novel approach to co‐design may differ from other studies, it was centred around engaging a diverse range of stakeholders, supporting shared decision‐making and applying an iterative, multistage design that allowed for flexibility and incorporation of additional feedback throughout. The use of consensus methods can help reduce dominance or conformity in group discussions [39] and support the development of prioritised solutions—an approach recognised as integral to effective co‐design [30]. That said, the multistage nature of the approach was resource‐intensive and time‐consuming, spanning 10 months between the initial scoping survey and final modified Delphi round. Having various stakeholder groups involved in the different processes potentially helped reduce the risk of fatigue from prolonged, repeated engagement in activities. Additionally, the planned 75% attendance at the consensus meetings was not always achieved. However, comprehensive meeting minutes were distributed to all, including those who were unable to attend, which provided an additional opportunity to contribute to the discussion. Meeting minutes were also confirmed by attendees, strengthening the validity of the process. Similarly, the response rate of the modified Delphi survey review by the study investigators was less than the planned 70%. However, in hindsight, some investigators were involved with the various aspects of the co‐design process or had solely analytical roles and therefore did not complete the survey, making the 70% not possible to achieve. An advantage of our co‐design study was that a small pilot stage was included to test the developed service intervention documents at one hospital with six participants. Feedback from this real‐world testing was important in further defining processes and scope of the follow‐up service intervention. Although three consultation pathways outlining the service steps and potential service coordinator activities had been identified, feedback from the reflection meeting post pilot testing led to the inclusion of additional pathways. For example, the service coordinator reported that they often only provided advice and education to participants, without onward referral. Therefore, an ‘advice and education’ pathway was included. The final service intervention will be tested in the feasibility randomised controlled trial of up to 100 participants, which will collect data on feasibility, clinical outcomes (e.g., health status, unmet needs, hospital readmissions and functional independence), cost implications, and fidelity to assess the service coordinators' role within the intervention [27]. The findings from this study will enhance the understanding of the service design, support integration into existing models of care and clarify cost and resource implications to inform a future fully powered trial assessing the intervention's effectiveness.

## 5 Conclusions

Data drawn from clinical quality registries can help close healthcare gaps, not only by providing outcome data, but also, potentially, by informing the provision of targeted interventions after hospitalisation for stroke. Using an iterative and multifaceted consensus‐based co‐design process, we have been able to design a new registry‐based hospital‐initiated follow‐up intervention for people living with stroke. A multisite feasibility randomised controlled trial is being completed in 2024 to assess the feasibility and outcomes of implementing this follow‐up service for people living with stroke.

## Author Contributions

**Tara Purvis:** methodology, data curation, formal analysis, writing – review and editing. **Andrew G. Ross:** methodology, investigation, data curation, formal analysis, writing – review and editing. **Jannette M. Blennerhassett:** methodology, writing – review and editing. **Karen M.Barclay:** methodology, data curation, formal analysis, writing – review and editing. **Tanya Frost:** methodology, writing – review and editing. **Dana Wong:** methodology, writing – review and editing. **Susan Hillier:** methodology, writing – review and editing. **Kathleen L. Bagot:** methodology, writing – review and editing. **Joosup Kim:** conceptualisation, funding acquisition, methodology, writing – review and editing. **Jennifer Cranefield:** methodology, writing – review and editing. **Katherine Jaques:** methodology, writing – review and editing. **Mark R. Nelson:** methodology, writing – review and editing. **Grant Russell:** methodology, writing – review and editing. **Colin Scott:** methodology, writing – review and editing. **Melita Stirling:** methodology, writing – review and editing. **Monique F. Kilkenny:** conceptualisation, funding acquisition,methodology, writing – review and editing. **Natasha A. Lannin:** methodology, writing – review and editing. **Timothy J. Kleinig:** conceptualisation, funding acquisition, methodology, writing – review and editing. **Rohan S. Grimley:** conceptualisation, funding acquisition, methodology, writing – review and editing. **Julie L. Morrison:** methodology, writing – review and editing. **Sandy Middleton:** conceptualisation, funding acquisition, methodology, writing – review and editing. **Vincent Thijs:** methodology, writing – review and editing. **Adele K. Gibbs:** methodology, data curation, writing – review and editing. **Dominique A. Cadilhac:** conceptualisation,methodology, funding acquisition, investigation, data curation, writing – original draft, writing – review and editing.

## Ethics Statement

Ethics approval for this project was provided by the Austin Health Human Research Ethics Committee; this included the site‐specific approval for the test site (HREC/86507/Austin‐2022). Approval for the use of the AuSCR data was provided by the AuSCR Research Task Group.

## Consent

Implied consent was used for the completion of all online surveys and the participation in the meetings/interviews.

## Conflicts of Interest

D.A.C. declares being the Data Custodian for the AuSCR. S.M. was Chair of the AuSCR Steering Committee and D.A.C., R.G., N.A.L. and M.F.K. are members of the AuSCR Management Committee. The other authors declare no conflicts of interest.

## Supporting information


**Supplemental Table A**: Demographics of scoping survey respondents.

## Data Availability

Data are not available as participants were informed of only non‐identifiable excerpts of their data being used in publications, and they did not consent to data sharing.
