# Supplementary material for: Co‐Design of a Registry‐Based Tailored Follow‐up Service Intervention for People Living With Stroke: A Multiple Method Consensus Approach
Source: Health Expect. 2025 Oct 29;28(6):e70474. doi: 10.1111/hex.70474 (PMC12569523; doi:10.1111/hex.70474)
Supplement: Supplementary file 1 — Supplemental Table A: Demographics of scoping survey respondents. [file HEX-28-e70474-s001.docx]

**Co-design** **of a registry-based tailored follow-up service intervention for people living with stroke: a multiple method consensus approach**

Tara Purvis, Andrew G Ross, Jannette M. Blennerhassett, Karen M. Barclay, Tanya Frost, Dana Wong, Susan Hillier, Kathleen L Bagot, Joosup Kim, Jennifer Cranefield, Katherine Jaques, Mark R Nelson, Grant Russell, Colin Scott, Melita Stirling, Monique F Kilkenny, Natasha A. Lannin,Timothy J Kleinig, Rohan S Grimley, Julie L Morrison, Sandy Middleton, Vincent Thijs, Adele K. Gibbs, Dominique A Cadilhac, on behalf of the A-LISTS investigator group

**Supplemental Material Inclusions:**

**Supplementary Table A: Demographics of scoping survey respondents**

**Scoping survey questionnaire**

**Modified Delphi survey**

**Working Group & Independent Group members**

**ACCORD (ACcurate COnsensus Reporting Document) guideline**

**COREQ (COnsolidated criteria for REporting Qualitative research) Checklist**

**Supplemental Table A:** **Demographics of scoping survey respondents**

|  | **Lived experience representative**  **N=8, n (%)*** | **Health service clinician/manager**  **N=28, n (%)*** | **Researcher/ academic**  **N=5, n (%)*** |
| --- | --- | --- | --- |
| **Gender** |  |  |  |
| Female | 6 (75) | 24 (86) | 4 (80) |
| **Age group**† |  |  |  |
| <30 years | 0 (0) | 2 (7) | 1 (20) |
| 30-39 years | 1 (14) | 4 (14) | 0 (0) |
| 40-49 years | 1 (14) | 11 (39) | 1 (20) |
| 50-59 years | 1 (14) | 8 (29) | 3 (60) |
| 60-69 years | 2 (29) | 3 (11) | 0 (0) |
| 70-79 years | 1 (14) | 0 (0) | 0 (0) |
| ≥80 years | 1 (14) | 0 (0) | 0 (0) |
| **Language spoken at home** |  |  |  |
| English | 8 (100) | - | - |
| **Clinical area**‡ |  |  |  |
| Emergency Department | - | 7 (25) | 0 (0) |
| Acute Hospital | - | 21 (75) | 1 (20) |
| Rehabilitation Hospital | - | 8 (29) | 0 (0) |
| Community-based facility | - | 3 (11) | 0 (0) |
| General Practice | - | 2 (7) | 0 (0) |
| Other^ | - | 1 (4) | 1 (20) |

^*^ % may not add to 100 due to rounding; †1 missing response for lived experience representative; ‡ more than one option able to be selected; ^ includes private practice, quality & safety unit

**Scoping survey questionnaire**

***Survey Section 1:*** Understanding who has completed our survey.

The following questions help us understand the characteristics of the people who completed the scoping survey and can help guide development of the clinical and research protocol.

**Clinicians [answer relevant to stakeholder group]**

- What is your professional background?

| Doctor | Nurse | Allied Health | Other, <free text> |
| --- | --- | --- | --- |

- Years working in your current role?
- What is your main specialty area? <free text>
- Where do you work? (tick all that apply)

| Emergency Dept | Hospital | Stroke Service | General Practice |
| --- | --- | --- | --- |
| Rehabilitation Hospital | | Community-based facility | |

Other, <free text>

- Do you or your health service participate in the Australian Stroke Clinical Registry?

| Yes | No | Unknown |
| --- | --- | --- |

- What is your age group?

| Less than 30 | 30-39 | 40-49 | 50-59 | 60-69 |
| --- | --- | --- | --- | --- |

- Are you?

| Female, woman | Male, man | Non-binary, gender diverse | Prefer not to say |
| --- | --- | --- | --- |
| Not listed, I identify as <free text> | |  | |

[For hospital-based clinicians who work within a Stroke Service]

- Is there a stroke unit at your hospital?

| Yes | No |
| --- | --- |

- Is there a stroke follow-up service at your hospital?

| Yes | No |
| --- | --- |

- If yes (follow-up service), please describe <free text> (prompts: who contacts discharged patients? What time period after discharge? Which patients post stroke are contacted (e.g., clinical criteria?)? What happens? Are you willing to share hospital protocol? How often are patients followed up? Are there any particular criteria for choosing who you follow up? )
- If yes (follow-up service), how could you do things differently to improve the service? <free text>
- If no (follow-up service), would a follow-up service be of value to patients with stroke beyond the first three months of recovery post their discharge from hospital? <free text>

**Patient representatives [answer relevant to stakeholder group] – people with lived experience of stroke or non-clinical respondents**

- What is your age group?

| Less than 30 | 30-39 | 40-49 | 50-59 | 60-69 | 70-79 | 80+ |
| --- | --- | --- | --- | --- | --- | --- |
|  |  |  |  |  |  |  |

- Are you?

| Female, woman | Male, man | Non-binary, gender diverse | Prefer not to say |
| --- | --- | --- | --- |
| Not listed, I identify as <free text> | |  | |

- In completing this survey who do you represent?

| Person with stroke | Carergiver | Advocacy organisation |
| --- | --- | --- |

Other, <free text>

- If you are a person with stroke, when was your last stroke? MM/YYYY
- Main language spoken at home?

| English | Other, list |
| --- | --- |

- Did you complete high school

| Yes | No | Prefer not to say |
| --- | --- | --- |

- Have you completed any education or training post high school?

| Yes | No | Prefer not to say |
| --- | --- | --- |

- If you present an advocacy organisation, please list name of the organisation <free text>
- What is your main reason for responding to this survey? <free text>

***Survey Section 2:***

Identifying patients eligible for a hospital-initiated stroke follow-up service. For the stroke follow-up service, we need to work out which people discharged from hospital six months after their stroke event would benefit most from having a follow-up consultation. The Australian Stroke Clinical Registry (AuSCR) collect information from people with stroke at three to six months post-stroke. We can use this information to identify those who may benefit most from additional support from stroke experts that treated them in hospital.

- In your opinion which people would be best targeted for a stroke follow-up service initiated by the treating hospital?  <free text>
- Who do you think should be involved? [e.g. medical, allied health or other professionals] <free text>
- What do you think might be barriers to providing such a service? <free text>
- What do you think might be offered to help make access to a service easier for patients? <free text>
- Other comments: <free text>

Feedback on methods of advising hospitals of discharged patients eligible for the follow-up service. Once we have identified people from the AuSCR follow-up survey data, a notification report will be provided to the hospital with details of each eligible patient based on certain criteria. We need to decide the details on that process.

An example draft report is provided below for your reference (not included in these Supplemental Materials). The final version will be developed by the A-LISTS trial Co-design Working Group.

[Health professionals working in acute hospital that treat stroke]

What is the best way to provide information about discharged patients with unmet needs to your hospital? When answering questions, think about what would work best at your hospital.

- Who should receive the notification? The report should be sent to (tick all that may apply):

|  | Head of Stroke Unit |
| --- | --- |
|  | AuSCR Hospital Coordinator (main person nominated to collect AuSCR data) |
|  | Stroke Coordinator |
|  | The person who is assigned and trained to initiate the stroke follow-up service |
|  | Tailored for each hospital; that is, each hospital nominates |
|  | Other, <free text> |

Unsure

- Method of notification

|  | Secure email to a monitored email inbox, role specific, not individual |
| --- | --- |
|  | Secure email to individual nominated by Head of Stroke Unit |
|  | Alert generated within the AuSCR data system sent to the main AuSCR Site Clinician |
|  | Tailored for each hospital; that is, each hospital nominates |
|  | Other, <free text> |
|  | Unsure |

Below is the proposed content of the Referral Report to be sent to hospitals that will test implementing the follow-up service (not included in Supplemental Materials). The information in the report is based on data that will be available from the AuSCR and any new information obtained at the time the patient consented to participate in the study.

- Are there any other details that could be relevant to include in the report? Please describe below: <free text>

***Survey Section 3:*** Steps undertaken by hospital-based service co-ordinator (clinical protocol).

The following Table outlines the proposed steps for the clinical protocol that will be initiated once the hospital receives the Referral Report (not included in Supplemental Materials).

- Do you have any comments you wish to make on the logic of each step and activities? Is there something you think should be done differently? <free text>
- When the follow-up service co-ordinator contacts the patient, what information is important for the co-ordinator to ask the patient? Please describe, <free text>.
- Would a template outlining these assessment steps (discussion, action, outcomes) with space to complete for each patient’s responses and actions to undertake be helpful?

| Yes | No | Not sure |
| --- | --- | --- |

Please explain, <free text>

- Would this template be considered useful to include in the medical record?

| Yes | No | Not sure |
| --- | --- | --- |

- If yes, is there anything in that needs to be included?  <free text>
- What would be barriers to including in the completed template in the hospital medical record? <free text>
- Should a copy of the template form be sent to the patient’s GP with their permission?

| Yes | No | Not sure |
| --- | --- | --- |

- A dedicated service co-ordinator will be recruited to implement and deliver the service at each participating hospital. What role or skills should the service co-ordinator have for successful implementation? <free text>
- Who else do you think should be involved in the initial consultation? <free text>
- Are there any policies or regulations that will need to be considered to implement this service at a hospital? <free text>
- Can you foresee any limitations to overcome for the stroke follow-up service to be a success? <free text>
- Is there anything that could be provided to the person with stroke to support their involvement in the follow-up service? *E.g., referral document information?* <free text>
- Is there anything else that you think is important for consideration in the design of the stroke follow-up service? <free text>

**Modified Delphi survey**

Thank you for taking the time to review the intervention package and provide feedback. The intervention package has been subject to a co-design process including a broad range of end users (clinicians, consumer representatives, allied health staff and researchers) to help us develop a clinical protocol and supporting documentation.

There are five main sections of the survey addressing the different areas of the proposed follow-up service:

1) Clinical protocol

2) A-LISTS participant referral report (sent to the service co-ordinator)

3) Service coordinator initial contact form

4) Consultation summary and key contact template form for participants

5) A-LISTS training manual for service coordinators

When considering your response to each section, think about what would work best for you, consider your hospital or service. We anticipate that it will take approximately 30 minutes to 1 hour to review the documents. This survey will remain open for completion until **xxx**.

Many thanks for your ongoing support.

You can download the A-LISTS project summary below if required (download not included in these Supplemental Materials)

- Q1. For the purposes of this task, please identify your role

| Clinical representative | Patient representative |
| --- | --- |

**SECTION 1:** Please review the clinical protocol and provide feedback. The clinical protocol outlines the proposed service steps and activities we envisage will occur from identifying the participants to the first follow-up call from the service co-ordinator.

A downloadable version of the clinical protocol is available with a summary provided below (download and summary not included in these Supplemental Materials).

- Do you agree with the content outlined in the clinical protocol?

| Yes | No | Unsure |
| --- | --- | --- |

- What adaptions might you suggest and why? <free text>
- Are there any other factors to consider? <free text>

**SECTION 2:** Please review the A-LISTS participant referral report and provide feedback. The A-LISTS participant referral report is the initial documentation the service co-ordinator will receive from the research team. This information is securely sent to the service co-ordinator via Cloudstor and is derived from data collected by the Australian Stroke Clinical Registry (AuSCR) and data obtained from the A-LISTS baseline assessment. The baseline assessment is performed by an AuSCR team member before the service co-ordinator contacts the participant.

A downloadable version of the A-LISTS participant referral report is available with a summary provided below (download and summary not included in these Supplemental Materials).

- Do you agree with the content outlined in the A-LISTS participant referral report?

| Yes | No | Unsure |
| --- | --- | --- |

- What adaptions might you suggest and why? <free text>
- Are there any other factors to consider? <free text>

**SECTION 3:** Please review the Service Co-ordinator Initial Contact Form and provide feedback. This is the document the service co-ordinator will use to perform the initial phone call to the participant.

A downloadable version of the Service Co-ordinator Initial Contact Form is available with a summary provided below (download and summary not included in these Supplemental Materials).

- Do you agree with the content outlined in the service co-ordinator initial contact form?

| Yes | No | Unsure |
| --- | --- | --- |

- What adaptions might you suggest and why? <free text>
- Are there any other factors to consider? <free text>

**SECTION 4:** Please review the key contacts template form and consultation summary for participants and provide feedback. This document will be sent to participants via email or mail at the end of the initial assessment phone-call.

A downloadable version of the Service Co-ordinator Initial Contact Form is available with a summary provided below (download and summary not included in these Supplemental Materials).

- Do you agree with the content outlined in the key contacts template and consultation summary form for participants?

| Yes | No | Unsure |
| --- | --- | --- |

- What adaptions might you suggest and why? <free text>
- Are there any other factors to consider? <free text>

**SECTION 5:** A-LISTS training manual. Please download the A-LISTS training manual or review the summary and provide feedback (download and summary not included in these Supplemental Materials).

- Do you agree with the content outlined in the training manual?

| Yes | No | Unsure |
| --- | --- | --- |

- What adaptions might you suggest and why? <free text>
- Are there any other factors to consider? <free text>

**Working Group & Independent Group members**

| ***Working group members*** |  |
| --- | --- |
| Natasha Lannin |  |
| Lisa Murphy |  |
| Jennifer Cranefield |  |
| Katherine Jacques |  |
| Louise Kelly |  |
| Sibilah Breen |  |
| Mark Nelson |  |
| Grant Russell |  |
| Susan Hillier |  |
| Colin Scott |  |
| Michelle Hiddleston |  |
| Emma Butler |  |
| Hayley Crute |  |
| Jodi Burford |  |
| Tanya Frost |  |
| Dana Wong |  |
| Brooke Parsons |  |
| Melita Stirling |  |
|  |  |
| ***Independent group members*** |  |
| Brendon Glenn |  |
| Rene Stolwyk |  |
| Karen Grindon-Ekins |  |
| Saran Chamberlain |  |
| Tala Olins-Miller |  |
| Eleanor Horton |  |

**ACCORD (Accurate Consensus Reporting Document) guideline**

| Item No. | Section | Checklist Item (*help text*) | Page No. |
| --- | --- | --- | --- |
| T1 | **Title** | Identify the article as reporting a consensus exercise and state the consensus methods used in the title.  *For example, Delphi or nominal group technique.* | Title page |
| I1 | **Introduction** | Explain why a consensus exercise was chosen over other approaches. | 5,6 |
| I2 |  | State the aim of the consensus exercise, including its intended audience and geographical scope (national, regional, global). | 6 |
| I3 |  | If the consensus exercise is an update of an existing document, state why an update is needed, and provide the citation for the original document. | N/A |
| M1 | **Methods**  Registration | If the study or study protocol was prospectively registered, state the registration platform and provide a link. If the exercise was not registered, this should be stated.  *Recommended to include the date of registration.* | 7 |
| M2 | Selection of SC and/or panellists | Describe the role(s) and areas of expertise or experience of those directing the consensus exercise.  *For example, whether the project was led by a chair, co-chairs or a steering committee, and, if so, how they were chosen. List their names if appropriate, and whether there were any subgroups for individual steps in the process.* | 10-12,14 |
| M3 |  | Explain the criteria for panellist inclusion and the rationale for panellist numbers. State who was responsible for panellist selection. | 9-12 |
| M4 |  | Describe the recruitment process (how panellists were invited to participate).  *Include communication/advertisement method(s) and locations, numbers of invitations sent, and whether there was centralised oversight of invitations or if panellists were asked/allowed to suggest other members of the panel.* | 8,9,11 |
| M5 |  | Describe the role of any members of the public, patients or carers in the different steps of the study. | 2,6,8,9,12, Table 2, Supplemental Table A |
| M6 | Preparatory research | Describe how information was obtained prior to generating items or other materials used during the consensus exercise.  *This might include a literature review, interviews, surveys, or another process.* | 7,8 |
| M7 |  | Describe any systematic literature search in detail, including the search strategy and dates of search or the citation if published already.  *Provide the details suggested by the reporting guideline PRISMA and the related PRISMA-Search extension.* | - |
| M8 |  | Describe how any existing scientific evidence was summarised and if this evidence was provided to the panellists. | 7, 8 |
| M9 | Assessing consensus | Describe the methods used and steps taken to gather panellist input and reach consensus (for example, Delphi, RAND-UCLA, nominal group technique).  *If modifications were made to the method in its original form, provide a detailed explanation of how the method was adjusted and why this was necessary for the purpose of your consensus-based study.* | 9-12 |
| M10 |  | Describe how each question or statement was presented and the response options. State whether panellists were able to or required to explain their responses, and whether they could propose new items.  *Where possible, present the questionnaire or list of statements as supplementary material.* | 10, 12, 14 |
| M11 |  | State the objective of each consensus step.  *A step could be a consensus meeting, a discussion or interview session, or a Delphi round.* | 7-14 |
| M12 |  | State the definition of consensus (for example, number, percentage, or categorical rating, such as ‘agree’ or ‘strongly agree’) and explain the rationale for that definition. | 10, 12, 14 |
| M13 |  | State whether items that met the prespecified definition of consensus were included in any subsequent voting rounds. | 10, 11, 12 |
| M14 |  | For each step, describe how responses were collected, and whether responses were collected in a group setting or individually. | 9-12, 14 |
| M15 |  | Describe how responses were processed and/or synthesised.  *Include qualitative analyses of free-text responses (for example, thematic, content or cluster analysis) and/or quantitative analytical methods, if used.* | 9, 10, 12-14 |
| M16 |  | Describe any piloting of the study materials and/or survey instruments.  *Include how many individuals piloted the study materials, the rationale for the selection of those individuals, any changes made as a result and whether their responses were used in the calculation of the final consensus. If no pilot was conducted, this should be stated.* | 7 |
| M17 |  | If applicable, describe how feedback was provided to panellists at the end of each consensus step or meeting.  *State whether feedback was quantitative (for example, approval rates per topic/item) and/or qualitative (for example, comments, or lists of approved items), and whether it was anonymised.* | 10, 11 |
| M18 |  | State whether anonymity was planned in the study design. Explain where and to whom it was applied and what methods were used to guarantee anonymity. | 8-12 |
| M19 |  | State if the steering committee was involved in the decisions made by the consensus panel.  *For example, whether the steering committee or those managing consensus also had voting rights.* | Methods |
| M20 | Participation | Describe any incentives used to encourage responses or participation in the consensus process.  *For example, were invitations to participate reiterated, or were participants reimbursed for their time.* | 6, 8,12 |
| M21 |  | Describe any adaptations to make the surveys/meetings more accessible.  *For example, the languages in which the surveys/meetings were conducted and whether translations or plain language summaries were available*. | 10-12 |
| R1 | Results | State when the consensus exercise was conducted. List the date of initiation and the time taken to complete each consensus step, analysis, and any extensions or delays in the analysis. | Figure 1, throughout methods |
| R2 |  | Explain any deviations from the study protocol, and why these were necessary.  *For example, addition of panel members during the exercise, number of consensus steps, stopping criteria; report the step(s) in which this occurred.* | - |
| R3 |  | For each step, report quantitative (number of panellists, response rate) and qualitative (relevant socio-demographics) data to describe the participating panellists. | 15-16, 18 |
| R4 |  | Report the final outcome of the consensus process as qualitative (for example, aggregated themes from comments) and/or quantitative (for example, summary statistics, score means, medians and/or ranges) data. | 19, Table 4, Figure 2 |
| R5 |  | List any items or topics that were modified or removed during the consensus process. Include why and when in the process they were modified or removed. | Tables 3-5, 16, 18-19 |
| D1 | Discussion | Discuss the methodological strengths and limitations of the consensus exercise.  *Include factors that may have impacted the decisions (for example, response rates, representativeness of the panel, potential for feedback during consensus to bias responses, potential impact of any non-anonymised interactions).* | 19, 22-23 |
| D2 |  | Discuss whether the recommendations are consistent with any pre-existing literature and, if not, propose reasons why this process may have arrived at alternative conclusions. | 19-21 |
| O1 | Other information | List any endorsing organisations involved and their role. | 25 |
| O2 |  | State any potential conflicts of interests, including among those directing the consensus study and panellists. Describe how conflicts of interest were managed. | 25 |
| O3 |  | State any funding received and the role of the funder.  *Specify, for example, any funder involvement in the study concept/design, participation in the steering committee, conducting the consensus process, funding of any medical writing support. This could be disclosed in the methods or in the relevant transparency section of the manuscript. Where a funder did not play a role in the process or influence the decisions reached, this should be specified.* | 25 |

From: PLoS Med 21(1): e1004326. <https://doi.org/10.1371/journal.pmed.1004326> For more information see: <https://www.ismpp.org/accord>

**COREQ (COnsolidated criteria for REporting Qualitative research) Checklist**

| **No.** | **Item** | **Guide questions/ description** | **Page** |  |
| --- | --- | --- | --- | --- |
| **Domain 1: research team and reflexivity** | | | | |
| Personal Characteristics | | | | |
| 1 | Interviewer/facilitator | Which author/s conducted the interview or focus group? | 10, 11, 13 |  |
| 2 | Credentials | What were the researchers credentials? *E.g. PhD, MD* | 10, 11, 13 |  |
| 3 | Occupation | What was their occupation at the time of the study | 10, 11, 13 |  |
| 4 | Gender | Was the researcher male or female? | 10, 11, 13 |  |
| 5 | Experience and training | What experience or training did the researcher have | 10, 11, 13 |  |
| Relationship with participants | | | |  |
| 6 | Relationship established | Was a relationship established prior to study commencement? | 10, 11, 13 |  |
| 7 | Participant knowledge of the interviewer | What did the participants know about the researcher? *E.g. personal goals, reasons for doing the research* | 10, 11, 13 |  |
| 8 | Interviewer characteristics | What characteristics were reported about the interviewer/facilitator? *E.g Bias, assumptions, reasons and interests in the research topic* | 10, 11, 13 |  |
| **Domain 2: study design** | | | |  |
| Theoretical framework | | | |  |
| 9 | Methodological orientation and theory | What methodological orientation was stated to underpin the study? *E.g. grounded theory, discourse analysis, ethnography, phenomenology, content analysis* | - |  |
| Participant selection | | | |  |
| 10 | Sampling | How were participants selected? *E.g. purposive, convenience, consecutive, snowball* | 9, 11, 13 |  |
| 11 | Methods of approach | How were participants approached? *E.g. face-to-face, telephone, mail, email* | 9, 11, 13 |  |
| 12 | Sample size | How many participants were in the study? | 9, Figure 1 |  |
| 13 | Non-participation | How many people refused to participate or dropped out? Reason? | 9 |  |
| Setting | | | |  |
| 14 | Setting of data collection | Where was the data collected? *E.g. home, clinic, workplace* | 9, 10, 11, 13 |  |
| 15 | Presence of non-participants | Was anyone else present besides the participants and researchers? | 9, 10, 11, 13 |  |
| 16 | Description of sample | What are the important characteristics of the sample? *E.g. demographic data, date* | 9, 10, 11, 13 |  |
| Data collection | | | |  |
| 17 | Interview guide | Were questions, prompts, guides provided to the authors? Was it pilot tested? | 10,13 |  |
| 18 | Repeat interviews | Were repeat interviews carried out? If yes, how many? | No |  |
| 19 | Audio/visual recording | Did the research use audio or visual recording to collect the data? | 10 |  |
| 20 | Field notes | Were field notes made during and/or after the interview of focus group | 10 |  |
| 21 | Duration | What was the duration of the interviews or focus group? | 9, 11 |  |
| 22 | Data saturation | Was data saturation discussed? | 22 |  |
| 23 | Transcripts returned | Were transcripts returned to participants for comment and/or correction? | 10, 11 |  |
| **Domain 3: analysis and findings** | | | |  |
| Data analysis | | | |  |
| 24 | Number of data coders | How may coders coded the data? | 10 |  |
| 25 | Description of the coding tree | Did authors provide a description of the coding tree? | 10, 13 |  |
| 26 | Derivation of themes | Were themes identified in advance or derived from the data? | 10, 13 |  |
| 27 | Software | What software, if applicable, was used to manage the data? | - |  |
| 28 | Participant checking | Did participants provide feedback on the findings? | Iterative process |  |
| Reporting | | | |  |
| 29 | Quotations presented | Were participant quotations presented to illustrate the themes/findings? Was each quotation identified? *E.g. participant number* | Throughout results |  |
| 30 | Data and findings consistent | Was there consistency between the data presented and the findings? | Throughout results |  |
| 31 | Clarity of major themes | Were major themes clearly presented in the findings? | Throughout results |  |
| 32 | Clarity of minor themes | Is there a description of diverse cases or discussion of minor themes? | Throughout results |  |

Developed from: Tong A, Sainsbury P, Craig J. Consolidated criteria for reporting qualitative research (COREQ): a 32-item checklist for interviews and focus groups. International Journal for Quality in Health Care. 2007. Volume 19, Number 6: pp. 349 – 357
